# Unveiling Valence State-Dependent Photocatalytic Water Splitting Activity and Photocathodic Behavior in Visible Light-Active Iridium-Doped BaTiO_3_

**DOI:** 10.1021/acsami.3c16710

**Published:** 2024-02-08

**Authors:** Sujana Chandrappa, Stephen Nagaraju Myakala, Namitha Anna Koshi, Simon Joyson Galbao, Seung-Cheol Lee, Satadeep Bhattacharjee, Dominik Eder, Alexey Cherevan, Dharmapura H. K. Murthy

**Affiliations:** † Department of Chemistry, Manipal Institute of Technology, Manipal Academy of Higher Education, Manipal 576104, Karnataka, India; ‡ TU Wien, Institute of Materials Chemistry, Getreidemarkt 9/BC/02, 1040 Vienna, Austria; § Indo-Korea Science and Technology Center (IKST), Korea Institute of Science and Technology, Bengaluru 560064, India; ∥ Center for Renewable Energy, Manipal Institute of Technology, Manipal Academy of Higher Education, Manipal 576104, Karnataka, India

**Keywords:** BaTiO_3_, Ir doping, photocatalytic water splitting, Ir valence state effect, photoelectrochemical H_2_ evolution

## Abstract

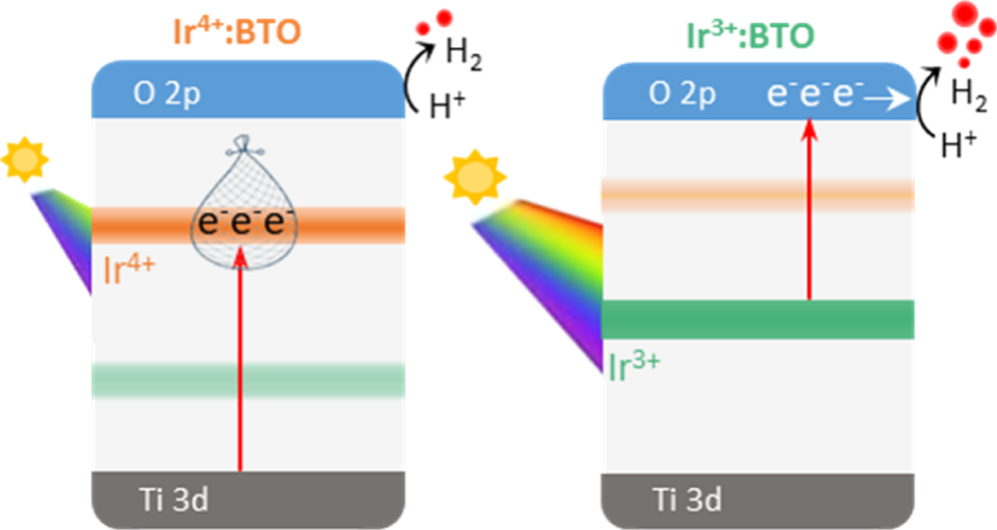

Despite having favorable energetics and tunable optoelectronic properties, utilization of BaTiO_3_ (BTO) for photocatalytic reactions is limited by its absorption only in the ultraviolet region. To address this challenge, BTO is doped with iridium (Ir) to induce visible light absorption. The visible light-induced photocatalytic H_2_ generation efficiency is enhanced by 2 orders of magnitude on selective conversion of the Ir valence state from Ir^4+^ to Ir^3+^. To understand such intriguing behavior, valence state-dependent changes in the optoelectronic, structural, and surface properties and electronic band structure are comprehensively investigated. The effect of electron occupancy change between Ir^4+^ (t_2g_^5^ e_g_^0^) and Ir^3+^ (t_2g_^6^ e_g_^0^) and their energetic positions within the band gap is found to significantly influence H_2_ generation. Besides this, converting Ir^4+^ to Ir^3+^ enhanced the photocathodic current and lowered the onset potential. Results aid in designing photocatalysts to efficiently use low-energy photons for enhancing solar H_2_ production in these emerging BTO-based photocatalysts. Collectively, the observations made in this work highlight the promising application of Ir^3+^:BTO in z-scheme photocatalysis.

## 1 Introduction

Due to their energetically favorable conduction band (CB) and valence band (VB) potentials, prolonged stability under an aqueous environment, compositional flexibility, and susceptibility to both anionic/cationic doping, ABO_3_-type titanate-based perovskite materials have emerged as promising photocatalysts for the water splitting reaction.^1−3^ Recent demonstration of H_2_ evolution via water splitting using 100 m^2^ photocatalyst modules comprising aluminum-doped SrTiO_3_ further demonstrated the potential of ABO_3_-type perovskites toward cost-effective and scalable solar fuel generation.^4^ Like SrTiO_3_, BaTiO_3_ (BTO) is another promising candidate to drive photocatalytic water splitting reactions. The choice of BTO is favored by the prospect of phase-dependent ferroelectric nature that can potentially promote charge generation yield and is thus expected to enhance the efficiency of the photocatalytic reaction.^5−7^

Despite these promising prospects and advantages, BTO can absorb only ultraviolet (UV) light due to its wide band gap (3.2 eV), which constitutes less than 5% of the solar spectrum.^8,9^ Considering the absorption cross-section and band gap of BTO, the theoretically possible maximum solar-to-hydrogen (STH) energy conversion efficiency would not exceed 2%.^10^ However, at least 10% STH energy conversion efficiency is required to make photocatalytic technology feasible for industrial applications. In this direction, extending the spectral response of BTO to enable maximum utilization of incoming sunlight is crucial. For this purpose, doping transition metal (TM) ions to the host lattice has been demonstrated to be a promising approach in various types of ABO_3_ perovskites.^8,11−16^ However, the observed photocatalytic H_2_ evolution rate for TM-doped perovskites could not always be correlated to the extent or magnitude of visible light absorption efficiency. For example, SrTiO_3_ doped with Cr, Rh, and Sb often showed inadequate H_2_ evolution compared to its undoped counterpart despite absorbing visible light.^12,14,17,18^ Thus, merely ensuring an extended absorption via doping does not necessarily enhance the photocatalytic H_2_ generation efficiency.

A careful examination of earlier reports revealed the key role of the dopant’s valence state and its electronic properties in determining the H_2_ generation efficiency. The valence state of Cr in Cr-doped SrTiO_3_ was primarily found to be 6+.^14^ However, converting Cr^6+^ to Cr^3+^ enhanced the photocatalytic H_2_ generation activity by six times.^17^ Similarly, Rh in Rh-doped SrTiO_3_ primarily existed as Rh^4+^, which showed virtually no photocatalytic activity.^15,18−20^ Converting Rh^4+^ to Rh^3+^ yielded factor three enhancement of photocatalytic H_2_ generation activity and also exhibited photoresponse up to λ ≤ 540 nm.^21^ To further understand the mechanism, the photoelectrochemical performance between Rh^4+^:SrTiO_3_ and Rh^3+^:SrTiO_3_ is compared.^22^ Rh^4+^:SrTiO_3_ showed a higher photocathodic current and a higher onset potential under visible light compared to Rh^3+^:SrTiO_3_. On the other hand, the valence state-dependent photoelectrochemical behavior of Ir-doped SrTiO_3_ was qualitatively different compared to Rh-doped SrTiO_3_. Ir^4+^:SrTiO_3_ showed a high photoanodic current under visible light, while Ir^3+^:SrTiO_3_ was inert.^23^ The observations discussed so far highlight the complexity in understanding valence state-dependent photocatalytic activity. A comprehensive correlation among the electronic nature of the dopant, its occupancy, and its energetic position within the forbidden region of the band gap with visible light-induced H_2_ generation efficiency is missing.

Among various metal dopants utilized, the choice of Ir can be understood by comparing its properties with those of well-studied Rh-doped SrTiO_3_. With Ir being a 5d element (Rh is 4d) in the periodic table, Ir-related impurity levels are located ∼0.5 eV above that of Rh levels within the band gap of SrTiO_3_.^23^ Consequently, Ir-doped SrTiO_3_ is expected to have optical absorption over a wider part of the solar spectrum, compared to Rh-doped SrTiO_3_. Indeed, pioneering work from the Kudo group realized extended optical absorption (λ ≤ 700 nm) in SrTiO_3_ and NaTaO_3_ upon Ir doping.^11,24^ The same group also investigated different methods of treating surface-loaded Ir (metallic) cocatalysts and their impact on the photocatalytic H_2_ evolution efficiency in Ir-doped SrTiO_3_.^11^ However, the effect of changing the valence state of Ir on the optoelectronic properties and/or photocatalytic activity is yet to be studied in detail. Thus, a further understanding of the intriguing effects of the Ir valence state on the photocatalytic performance would aid in the effective utilization of low-energy photons for H_2_ generation, which is still a challenge.

Most of the earlier studies on dopant-induced photocatalytic activity are conducted on SrTiO_3_. Despite the advantages and unique prospects of BTO, a few reports on visible light-induced photocatalytic activity in BTO are reported.^8,25,26^ Rh doping-induced p-type behavior in BTO was reported by Maeda et al. almost a decade ago.^8^ Recently, Shi et al. investigated the effect of the Rh doping level on the Fermi level position and n- to p-type transition was studied by isolating the cathodic contribution by the Rh dopant.^27^ Despite years of research related to BTO as a H_2_ evolution photocatalyst, there exists no report investigating the effect of the dopant’s valence state on the optoelectronic properties and subsequent photocatalytic and/or photoelectrochemical behavior of BTO.

In this direction, Ir-doped BTO as a promising visible light-absorbing p-type material has been recently developed for the first time.^16^ The current work reveals valence state-dependent differences in the optoelectronic/photophysical properties and their influence on the photoelectrochemical behavior and photocatalytic H_2_ evolution efficiency in Ir-doped BTO. Detailed analysis using a range of complementary tools offers insights into photocatalyst design for effectively harvesting low-energy photons to generate H_2_.

## 2 Materials and Methods

### 2.1 Photocatalyst Synthesis

Barium nitrate (99%, Merck) and titanium(IV) oxide nanopowder (NanoArc. anatase 99%, Alfa Aesar) and iridium(IV) oxide (99.9%, Aldrich) were used for synthesis without any further treatment. 2 mol % Ir-doped BTO is synthesized according to the earlier report and was named as Ir^4+^:BTO.^16^ As obtained, Ir^4+^:BTO was subjected to heat treatment under a reducing atmosphere and is referred to as Ir^3+^:BTO (detailed methodology provided below). For comparison, undoped BTO was also synthesized according to the earlier report.^16^

Ir^4+^:BTO was reduced by a temperature-programmed reduction (TPR) technique under a H_2_ flow using a Belcat II (BEL Japan) instrument. Prior to the reduction process, the sample was treated in an Ar gas flow (50 mL min^–1^) at 300 °C, followed by cooling to 50 °C. Next, the sample was subjected to a reducing environment with 5% H_2_ in He at a flow rate of 30 mL min^–1^, which was ramped to 400 °C at 10 °C min^–1^ rate and held at this temperature for 1 h.

### 2.2 Photodeposition of the Pt Cocatalyst and Photocatalytic H_2_ Evolution Reaction

The H_2_ evolution experiments were performed in a slurry-type reactor illuminated from a side by using a 200 W Hg lamp (Superlite I 05, Lumatec), which acted as a source for both visible light (λ = 400–700 nm, total power of 500 mW) and UV light (λ = 240–400 nm, total power of 175 mW) experiments. The spectral distribution of the Hg lamp is shown in Figure S1. The experiments were carried out in a batch-type mode with the reaction solution kept at 15 °C under constant stirring at 500 rpm throughout the reaction time. In a single run, 100 mg of the photocatalyst was added to 40 mL of 10 vol % aqueous methanol solution and ultrasonicated for 5 min to obtain a homogeneous suspension. The suspension was then transferred to the reactor and purged for 10 min with argon gas at 30 mL min^–1^ to remove dissolved oxygen from the reaction solution. Before closing the reactor, the amount of aqueous H_2_PtCl_6_ solution corresponding to the 1 wt % of Pt with respect to the photocatalyst amount was added as a precursor for in situ photodeposition of Pt, which acted as a H_2_ evolution reaction cocatalyst. During light irradiation, the reactor was kept airtight. After 24 h of illumination, 200 μL of the gaseous sample was taken from the headspace of the reactor and analyzed using gas chromatography (Shimadzu GC-2030 equipped with a barrier discharge ionization detector). A 6-point calibration profile was used to accurately quantify the amount of H_2_ evolved and translate it to mole values presented in the main text.

### 2.3 Photoelectrochemical Measurements

Electrodes for the photoelectrochemical measurements were prepared as follows. 2.5 mg of photocatalyst (Ir^4+^:BTO and Ir^3+^:BTO) was added to the solution containing 120 μL of distilled water, 100 μL of isopropanol, 5 μL of dimethylformamide, and 5 μL of Nafion. The solution was sonicated for 45 min to prepare the photocatalyst ink. 20 μL of the prepared ink was drop-cast on the ITO (indium tin oxide)-coated glass substrate (resistance of <6 ohm/sq). The apparent area of the electrode containing the ink was 0.25 cm^2^ area. The electrodes were dried under an infrared lamp and calcined at 300 °C for 3 h. A potentiostat (Metrohm Autolab PGSTAT 204) with a three-electrode system with platinum as the counter electrode, reversible hydrogen electrode (RHE) as the reference electrode, and ITO-coated glass substrates as the working electrode was used for photoelectrochemical measurements. 0.1 M K_2_SO_4_ was used as an electrolyte. The current–voltage response was recorded at the scan rate of 25 mV s^–1^, and the chronoamperometric measurements were recorded at −0.2 V vs RHE. A solar simulator (Holmarc, HR-SS300WRM1-100A) equipped with an air mass 1.5 filter and a 300 W xenon short arc lamp (Ushio Inc. Japan) was the light source. The light coming from the xenon arc lamp was passed through a 420 nm long pass filter before illumination of the sample.

### 2.4 Structural, Surface, and Optoelectronic Characterization Tools

The powder X-ray diffraction (XRD) was performed using a Bruker D_2_ Phaser using Cu Kα radiation as the radiation source of wavelength λ = 1.54 Å. The samples were mounted on the sample holder, and the XRD pattern was recorded from 20 to 90° 2θ with 0.02° 2θ step size and a scan speed of 0.3 s per step.

The optical absorption of the samples was measured using a UV/vis/NIR (near-infrared) spectrometer (PerkinElmer lambda 750) in diffuse reflectance mode, equipped with an integrating sphere.

Valence states of the constituting elements of the material synthesized were investigated by X-ray photoelectron spectroscopy (XPS) (Thermo-Scientific NEXSA) using Al Kα (1486.6 eV) as the X-ray source. The operating pressure was in the ultrahigh-vacuum range from 10^–8^ to 10^–10^ mbar. The XPS survey spectra were recorded at a pass energy of 200 eV with a step size of 1 eV. The XPS higher energy resolution spectra were recorded with a pass energy of 50 eV with a step size of 0.1 eV. A flood gun was used to eliminate the charging effects. The spectral charge correction was performed using carbon with a C 1s peak appearing at 284.8 eV. Ultraviolet photoelectron spectroscopy (UPS) or valence band-XPS (VB-XPS) was performed using a spectrometer (Thermo-Scientific NEXSA) with a He I (21.22 eV) excitation source. The pass energy used was 2 eV with 0.050 eV step size. The operating pressure was in the ultrahigh-vacuum range from 10^–8^ to 10^–10^ mbar. The VB spectra of Ir^4+^:BTO and Ir^3+^:BTO were plotted by converting the counts versus kinetic energy (eV) to counts versus binding energy (eV). Kinetic energy to binding energy conversion was performed using the equation, photon energy = binding energy + kinetic energy, considering that the spectrometer is calibrated such that the Fermi energy will appear at binding energy zero. Due to the sensitivity of the XPS instrument, BTO doped with 3 mol % of Ir was essential to better visualize the differences in the changes in the valence state of Ir (after H_2_-TPR experiments) in the Ir 4f peak binding energy.

The surface area of the photocatalysts was determined using a Belsorb (II) mini-instrument (BEL, Japan). The N_2_ sorption analysis was performed at 77 K to measure the Brunauer–Emmet–Teller (BET) specific surface area.

### 2.5 Computational Method to Analyze the Electronic Structure

Computational investigations were undertaken using density functional theory (DFT). All spin-polarized calculations are performed using the projector-augmented wave (PAW) method implemented in the Vienna Ab initio Simulation Package (VASP) with the Perdew–Burke–Ernzerhof (PBE) formalism of generalized gradient approximation (GGA) to account for exchange correlations.^28−32^ The electron wave function is expanded in plane waves with a cutoff energy of 500 eV. The cell and atomic relaxations are carried out with the energy and force convergence criteria set to 10^–6^ eV and −0.01 eV/Å, respectively. For the simulation of Ir doping in BTO, a 135-atom supercell with a Monkhorst–Pack *k*-mesh of 3 × 3 × 3 is used to sample the Brillouin zone for geometry optimization and appropriate denser mesh is used to calculate the density of states. Note that Ir is doped at the Ti site in the BTO cell. Since band gaps are not quantified correctly by GGA, we employ the rotationally invariant approach of GGA + *U*, where *U* values of 4 and 8 eV are used for Ti and O, respectively. For Ir, a *U* value of 2 eV is used in the present work. The Ir valence state-dependent optical absorption spectra are calculated by converting the complex dielectric function obtained from the VASP to the absorption coefficient. To determine the optical response of Ir-doped BTO, the frequency-dependent complex dielectric function ε(ω) = ε_1_(ω) + iε_2_(ω) has been calculated, which indicates the linear response of material to the electromagnetic field. The electronic structure and dielectric function are related to each other. Here, the imaginary part of dielectric function ε_2_(ω) is given by the summation over the large number of empty states and the real part ε_1_(ω) is obtained from ε_2_(ω) using Kramers–Kronig relation. Since the absorption range of the solar spectrum is crucial in optoelectronic materials, the optical absorption coefficient α(ω) is calculated using the following relation
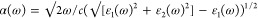


The spectra of absorption coefficient vs wavelength are obtained by taking the average of three principal non-zero diagonal constituents (α*_xx_*(ω), α*_yy_*(ω), and α*_zz_*(ω) corresponding to the *a*, *b*, and *c* axes, respectively).

## 3 Results and Discussion

The first part will discuss the controlled transformation of the Ir valence state from Ir^4+^ to Ir^3+^ and the resulting optoelectronic/structural/surface properties. The latter part will focus on elucidating valence state-dependent photoelectrochemical behavior and photocatalytic H_2_ evolution activity.

Typically, the valence state of the dopant in ABO_3_-type titanate-based perovskites is manipulated by heating the sample under reducing conditions, for example, in the presence of H_2_ gas.^11,17,33^ In this approach, obtaining precise information on the selectivity toward a given reducible species is rather complex, i.e., it is difficult to find out whether the constituents of the perovskite host or the dopant is reduced. Hence, in the current work, the valence state of the Ir dopant from Ir^4+^ to Ir^3+^ is selectively converted by performing the H_2_-TPR experiment. In this method, the as-prepared Ir^4+^:BTO is subjected to a linear heating process in an enclosed furnace with a H_2_ flow. The difference in the reducing gas (H_2_) concentration before and after the reduction reaction is monitored by thermal conductivity detector (TCD) signal vs temperature. The H_2_-TPR experiment offers control/selectivity toward the reduction reaction and provides unique information to identify the chemical/electronic nature of the species reduced, unlike typically employed uncontrolled heating of the sample.^34^

**Figure 1:**
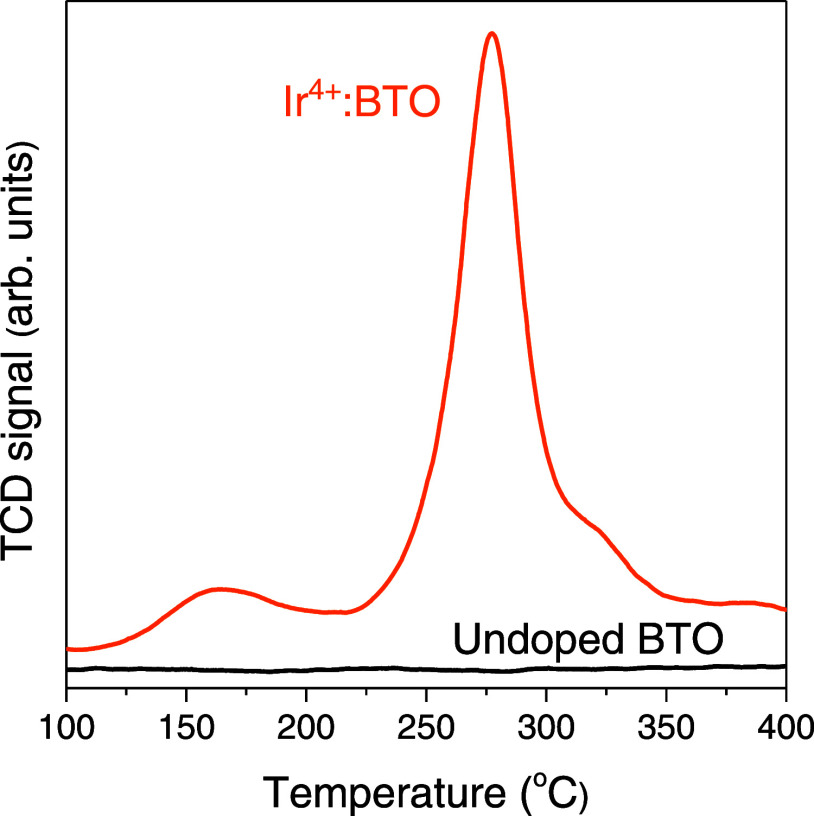
H_2_-TPR profiles of undoped BTO and 3 mol % doped Ir^4+^:BTO from 100 to 400 °C.

Figure 1 compares the H_2_-TPR profiles of Ir^4+^:BTO and undoped BTO. The presence of a distinct TCD signal response for Ir^4+^:BTO, which is virtually absent for undoped BTO, indicates that Ir-related species are being reduced during the H_2_-TPR experiment. Such comparison allowed us to rule out the possibility of the H_2_-TPR experiment reducing the constituents of host BTO lattice. Furthermore, the magnitude of the TCD signal (Figure S2) concomitantly decreased for samples with a lower Ir doping level. To further elucidate the electronic nature of the reduced Ir-related species, the H_2_-TPR experiment with IrO_2_ was conducted (Figure S3). Note that IrO_2_ was employed as the precursor to synthesize Ir-doped BTO that yielded a high concentration of Ir^4+^ species.^16^ On comparing H_2_-TPR profiles of Ir^4+^:BTO and IrO_2_, a similarity in the temperature range is noticed. Thus, the appearance of the peak in the H_2_-TPR profile of Ir^4+^:BTO is due to the reduction of Ir^4+^ to its lower valence state.

**Figure 2:**
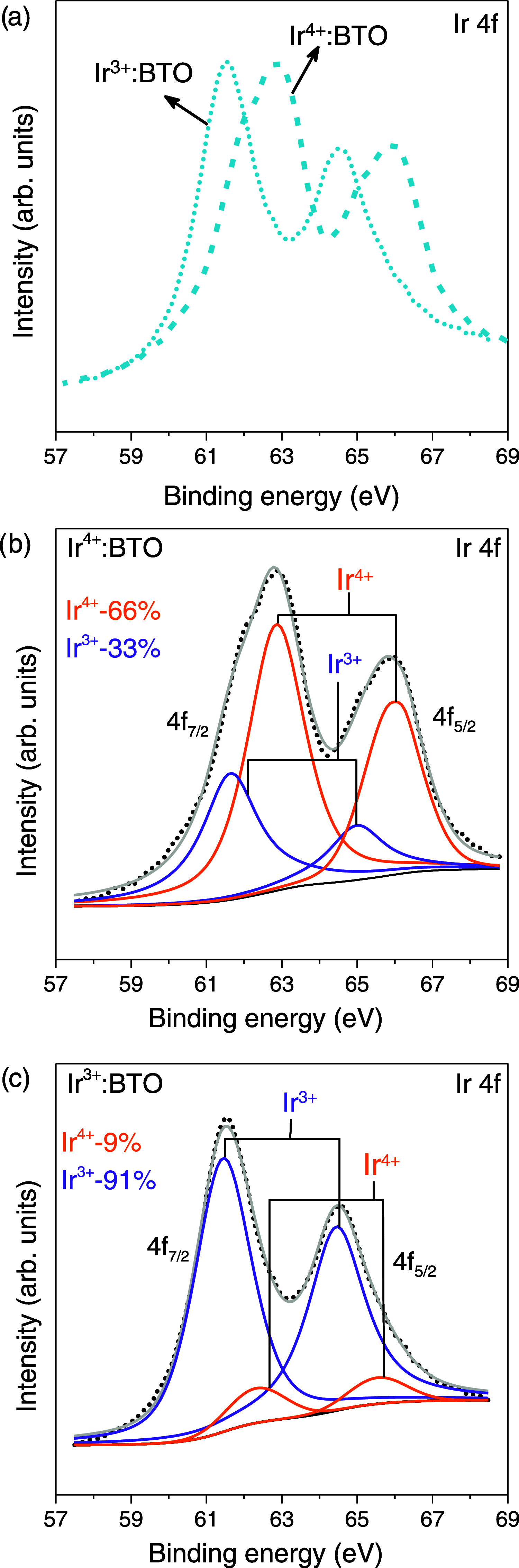
(a) Comparing the shift in Ir 4f core level spectra of Ir^4+^:BTO and Ir^3+^:BTO, corresponding to before and after the H_2_-TPR experiment. Deconvoluted high-resolution Ir 4f core level spectra of (b) Ir^4+^:BTO and (c) Ir^3+^:BTO.

To confirm the valence state of Ir^4+^ after the H_2_-TPR experiment, an XPS analysis is conducted. Figure 2 collectively presents the Ir 4f core level spectra of as-prepared Ir^4+^:BTO (before the H_2_-TPR experiment) and Ir^3+^:BTO obtained after the H_2_-TPR measurement. Figure 2a shows ≈1.3 eV shift toward lower binding energy for Ir^3+^:BTO compared to Ir^4+^:BTO. However, the binding energy shift for Ti 2p and Ba 3d is <0.1 eV (Figure S4), suggesting a high selectivity toward Ir-related reducible species during the H_2_-TPR experiment. This observation also agrees with the observation of a virtually absent TCD signal for undoped BTO in Figure 1.

Figures 2b and 2c depict the deconvoluted Ir 4f core level spectra of Ir^4+^:BTO before and after the H_2_-TPR experiment, respectively. The Ir 4f_7/2_ peak at ≈61.5 eV indicated in purple is characteristic of Ir in the 3+ valence state, while the peak at ≈62.5 eV indicated in orange corresponds to Ir in the 4+ valence state.^23^ Ir^4+^:BTO showed a high percentage (66%) of Ir^4+^. The concentration of Ir^3+^ increased from 33 to 91% after the H_2_ TPR experiment, whereas the Ir^4+^ content concomitantly decreased from 66 to 9%, and this sample is referred to as Ir^3+^:BTO. Such a significant increase in Ir^3+^ concentration is attributed to the selective conversion of Ir^4+^ to Ir^3+^ during the H_2_-TPR experiment. It is essential to note the absence of an XPS peak around 60.8 eV corresponding to Ir^0^ (metallic Ir)^35^ after H_2_-TPR experiments (Figure 2c).

**Figure 3:**
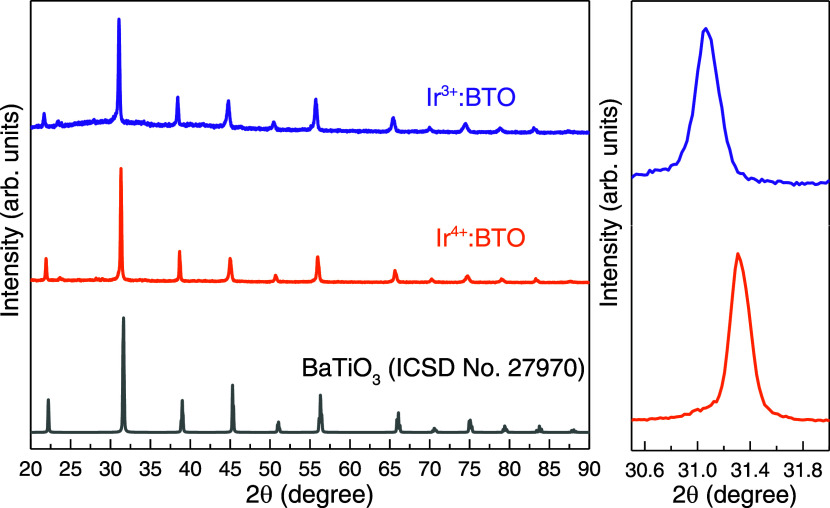
XRD patterns of Ir^4+^:BTO, Ir^3+^:BTO, and undoped BTO from the ICSD database.

XPS can primarily probe the changes in the valence state of the elements and dopants located on the surface. Note that the reduction method using the H_2_-TPR experiment is expected to induce changes in the bulk of the photocatalyst as well. To understand such a behavior, XRD and optical properties are investigated. Figure 3 compares the XRD patterns of Ir^4+^:BTO and Ir^3+^:BTO. By comparing with the database corresponding to undoped BTO (ICSD 27970), the cubic phase is retained for Ir^3+^:BTO, and the overall structural integrity is maintained. As discussed earlier, the H_2_-TPR experiment successfully converted a majority of the Ir^4+^ in the as-synthesized Ir-doped BTO sample to Ir^3+^. Note that Ir^3+^ (82 pm) has a greater ionic radius than does Ir^4+^ (76.5 pm). Considering the Ir dopant occupying Ti sites of the BTO host lattice,^16^ the conversion of Ir^4+^ to Ir^3+^ is expected to result in a lattice expansion. As expected, a shift toward a lower diffraction angle by ≈0.27° 2θ for Ir^3+^:BTO compared to Ir^4+^:BTO is observed. This observation indicates that the Ir valence state change after H_2_-TPR is not just on the surface but rather a bulk phenomenon.

**Figure 4:**
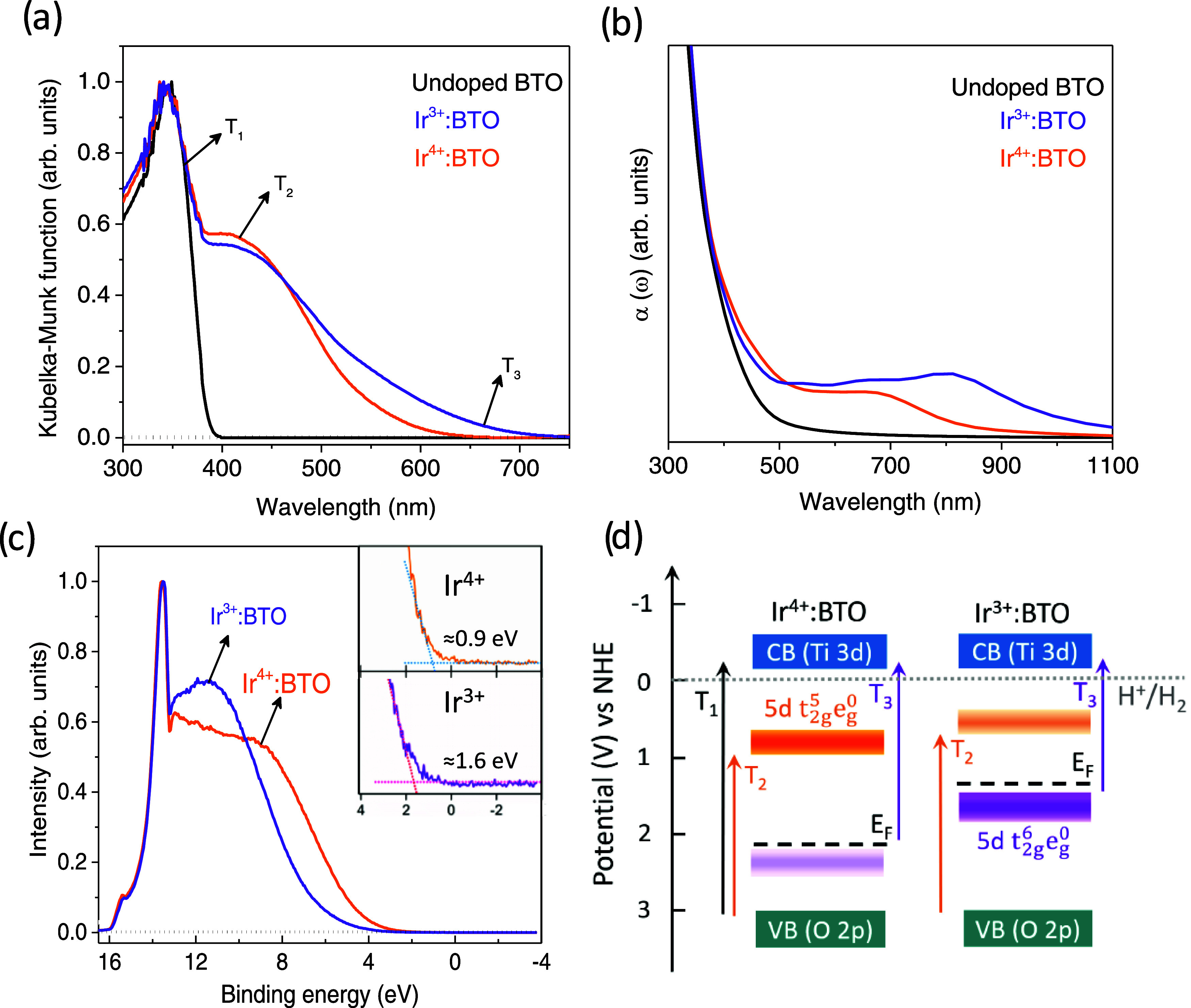
(a) Optical absorption spectra of undoped BTO, Ir^4+^:BTO, and Ir^3+^:BTO measured in diffuse reflection mode. (b) Computationally derived absorption coefficient values of undoped BTO, Ir^4+^:BTO, and Ir^3+^:BTO. (c) VB spectra of Ir^4+^:BTO and Ir^3+^:BTO and the inset shows the zoomed-in view from +4 to −4 eV. (d) Valence state-dependent band energy diagram for Ir^4+^:BTO and Ir^3+^:BTO showing the possible optical transitions, energetics involved, and electron occupancy.

Understanding the valence state-dependent optical properties offers insights into the electron occupancy and energetic position of the dopants in the forbidden region of the band gap. Figure 4a presents the optical absorption spectra of undoped BTO, Ir^4+^:BTO, and Ir^3+^:BTO. Besides the fundamental transition (T_1_ in Figure 4d) from the O 2p orbitals of VB to Ti 3d of CB, a broad absorption from 390 to 600 nm noticed for Ir^4+^:BTO (T_2_ in Figure 4d) is attributed to the electron transitions from VB to Ir^4+^ (Ir 5d t_2g_^5^ e_g_^0^ configuration) in-gap energy levels. Due to the partially occupied d^5^ electronic configuration of Ir^4+^, optical transitions from Ir^4+^ to the CB are unlikely. Let us now focus on the optical absorption of Ir^3+^:BTO, which is clearly found to be further red-shifted to 720 nm compared to Ir^4+^:BTO. The extended tail absorption from around 600 to 720 nm can be attributed to the electron transitions from Ir^3+^ (Ir 5d t_2g_^6^ e_g_^0^ configuration) to the CB (T_3_ in Figure 4d). Thus, for Ir^3+^: BTO, visible light photons can populate electrons in the CB, which is expected to improve the photocatalytic H_2_ evolution.

The effect of the Ir valence state on the optical absorption property is further investigated via the computational approach. Figure 4b depicts the absorption coefficient vs wavelength plot of undoped BTO, Ir^4+^:BTO, and Ir^3+^:BTO. Ir^4+^:BTO is obtained by substituting Ir for Ti sites of the BTO host.^16^ By adding one more electron to Ir^4+^, Ir^3+^ in BTO is modelled for the analysis. Ir-doped BTO photocatalysts exhibit extended optical absorption in the visible/NIR region compared to undoped BTO absorbing only in the UV region. The absorption of Ir^3+^:BTO extends beyond that of Ir^4+^:BTO toward the NIR region, owing to the pronounced T_3_ transition indicated in Figure 4d. Thus, the computational analysis of the electronic structure also shows a similar trend in valence state-dependent optical absorption properties and is in qualitative agreement with Figure 4a.

Figure 4c compares the VB spectra of Ir^4+^:BTO and Ir^3+^:BTO obtained from UPS. This experiment aims to understand how a change in the Ir valence state impacts the electronic band structure. A change in the electron occupancy upon conversion of Ir^4+^ to Ir^3+^ is expected to influence the density of states (DOS) and the Fermi level (*E*_F_) position. Indeed, the observed ≈0.9 eV shift toward higher binding energy corresponding to the spectral onset for Ir^3+^:BTO compared to Ir^4+^:BTO indicates an upward shift in the *E*_F_ position for Ir^3+^:BTO. The overall shape of the VB spectra changes, suggesting an increase in the DOS of Ir^3+^ states for Ir^3+^:BTO compared to Ir^4+^:BTO. Conclusions from Figure 4c agree well with the valence state-dependent electronic structure analysis (Figure S5) elucidated using the computational approach.

Figure 4d presents a band energy diagram based on the data discussed earlier. The VB maximum for most of the metal oxides is considered to be situated approximately at +3 eV vs the normal hydrogen electrode (NHE).^36^ Considering the band gap of undoped BTO as 3.2 eV, the absorption onsets of Ir^4+^:BTO and Ir^3+^:BTO, and the *E*_F_ position derived from the VB spectra, the band energy diagram is deduced. By virtue of its occupancy, Ir^4+^ levels located in the in-gap region can potentially act as electron traps. Considering the energetic position of Ir^4+^ levels with the potential required to reduce the H^+^ of water, an inadequate thermodynamic driving energy to generate H_2_ can be anticipated. However, after the H_2_-TPR experiment, the density of Ir^3+^ levels is increased, thus promoting the T_3_ transition. As a result, electrons can populate the CB, and they have sufficient energy to drive the H_2_ generation. Therefore, the valence state of the Ir dopant is found to significantly impact optical, electronic, structural, and surface properties. The following part of the Results and Discussion will reveal valence state-dependent photoelectrochemical behavior and photocatalytic H_2_ evolution activity.

### 3.1 Effect of the Ir Valence State on the Photoelectrochemical Behavior

**Figure 5:**
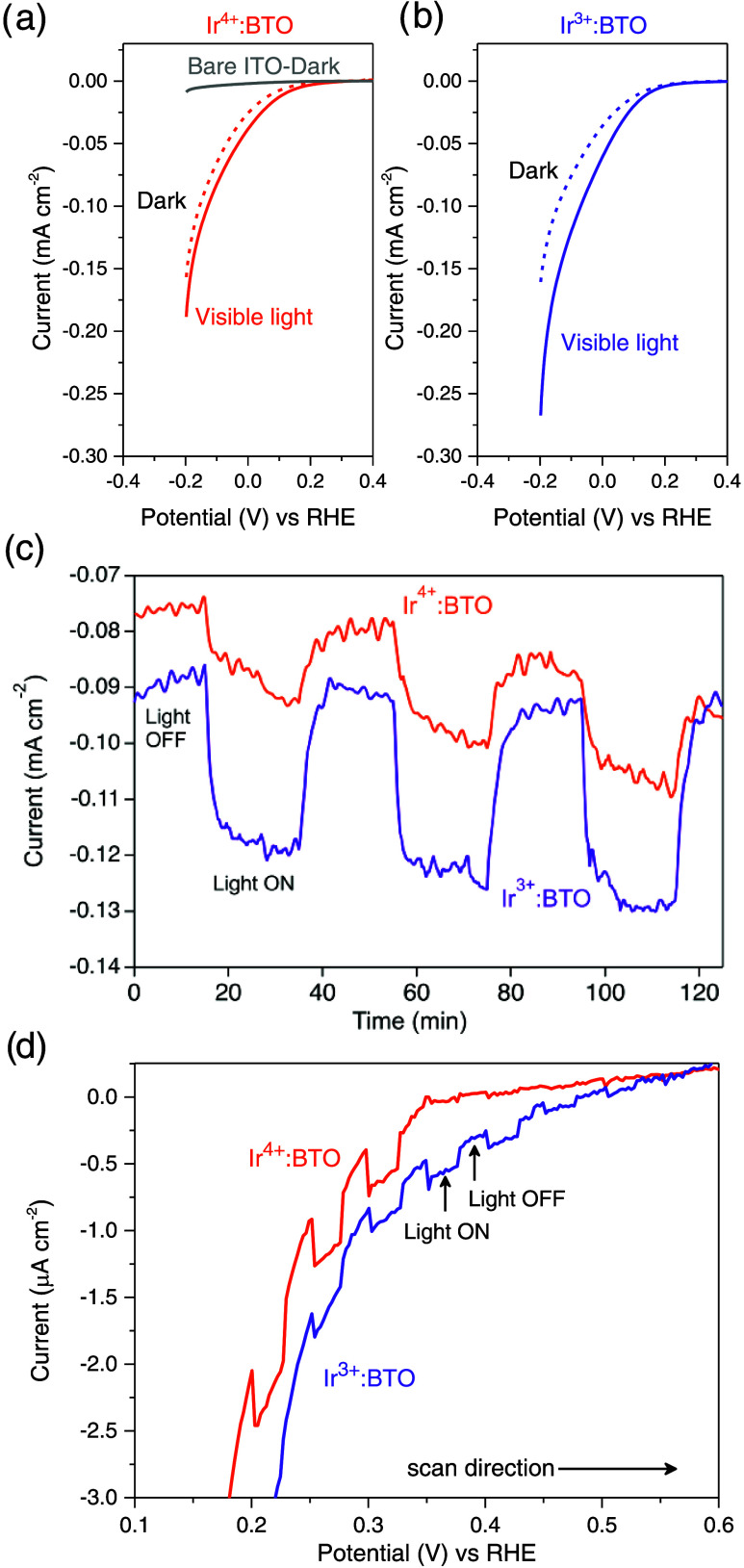
Linear sweep voltammograms (LSV) recorded in the dark and under visible light (λ > 420 nm) irradiation for (a) Ir^4+^:BTO and (b) Ir^3+^:BTO. (c) Chronoamperometric measurements of Ir^4+^:BTO and Ir^3+^:BTO conducted under intermittent visible light (λ > 420 nm) irradiation. (d) Comparing LSVs between Ir^4+^:BTO and Ir^3+^:BTO under intermittent visible light (λ > 420 nm) irradiation.

As discussed earlier, converting Ir^4+^ to Ir^3+^ in Ir-doped BTO has a significant influence on the optical absorption and the electronic band structure. To further probe these phenomena, the effect of the Ir valence state on the photoelectrochemical behavior is investigated by conducting linear sweep voltammetry, and the corresponding voltammograms (LSV) are presented in Figure 5. Figures 5a and 5b depict LSV of Ir^4+^:BTO and Ir^3+^:BTO, respectively, recorded in the dark and under visible light (λ > 420 nm) irradiation. For comparison, the LSV of bare ITO performed in the dark is provided in Figure 5a and shows a negligible response. Comparing the respective LSVs, photoinduced enhancement in the current is higher (light vs dark) for Ir^3+:^BTO compared to Ir^4+^:BTO. At −0.2 V vs RHE; Ir^3+^:BTO shows a factor of ≈1.7 increment in the photocurrent than Ir^4+^:BTO. Similar differences in valence state-dependent photoelectrochemical behavior are observed for Ir-doped BTO with a higher doping level as well (Figure S6). Furthermore, visible light-induced enhancement in the cathodic current is supported by chronoamperometric studies performed at −0.2 V vs RHE, depicted in Figure 5c. Observing a negative photocurrent for both Ir^3+^:BTO and Ir^4+^:BTO is in accordance with the LSVs discussed earlier. Moreover, both the photocatalysts exhibit a nondecaying photocurrent response in the time window measured, highlighting their stability during the visible light irradiation. A factor of ≈1.6 increment in the photocurrent observed for Ir^3+^:BTO compared to Ir^4+^:BTO indicates its utility in photocatalytic reactions. The observed increment in photocurrent during the chronoamperometry is further validated by monitoring the H_2_ evolved as illustrated in Figure S7. An increment of ≈2.5 times in H_2_ evolution recorded for Ir^3+^:BTO with concurrent enhancement in Faradaic efficiency (calculated at the end of 4 h, refer the Supporting Information) by a factor of ≈2.3 times relative to Ir^4+^:BTO clearly demonstrates the Ir valence state effect on the photoelectrochemical activity. Figure 5c compares the photocurrent onset between Ir^3+^:BTO and Ir^4+^:BTO recorded under intermittent visible light irradiation. A minimum of ≈0.2 V shift in the onset potential toward a lower cathodic side is noticed for Ir^3+^:BTO compared to Ir^4+^:BTO. A similar valence state-dependent shift in the onset potential was observed in Rh-doped SrTiO_3_.^22^

In short, the following information can be deduced through a collective analysis of Figure 5. A higher photocurrent magnitude with enhanced Faradaic efficiency accompanied by a lower onset potential corroborates the positive effect of extended light absorption and electron occupancy of the Ir dopant within the forbidden band gap region of Ir^3+^:BTO. These observations further aided in establishing the role of the Ir valence state in the optoelectronic properties and electronic band structure; both favor the applicability of Ir^3+^:BTO for the H_2_ evolution reaction. Hence, the visible light-induced enhancement in cathodic response paired with lower onset potential highlights the promising application of the Ir^3+^:BTO photocathodic material toward *z*-scheme photocatalysis.

### 3.2 Effect of the Ir Valence State on the Photocatalytic H_2_ Evolution Activity

**Figure 6:**
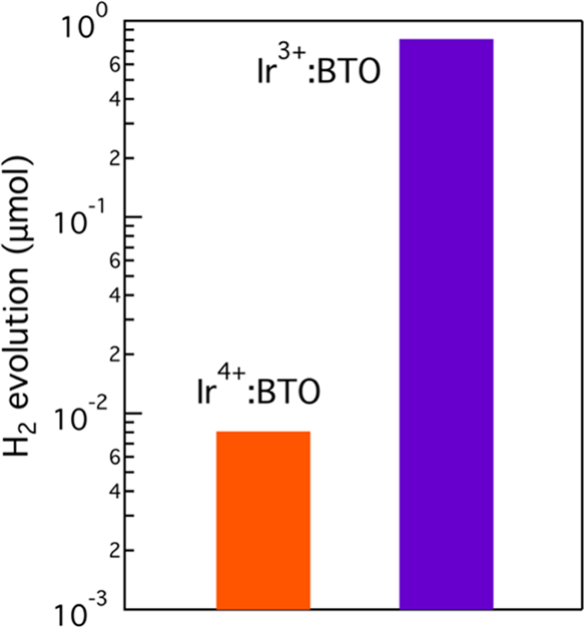
Valence state-dependent H_2_ evolution by 2 mol % Ir-doped BTO photocatalysts under visible light (λ > 400 nm). Reaction conditions: 1 wt % Pt cocatalyst loading, 100 mg of photocatalyst, 10 vol % aqueous methanol as hole scavenger, and 24 h.

Figure 6 depicts the effect of the Ir valence state on the photocatalytic H_2_ evolution activity under visible light (λ > 400 nm). Ir^3+^:BTO shows 2 orders of magnitude enhancement in H_2_ evolution compared to Ir^4+^:BTO. Note that it is essential to rule out that the H_2_-TPR measurement that converted Ir^4+^ to Ir^3+^ did not drastically alter the surface area. The BET surface area measurements (Table S1) after H_2_-TPR showed an increment from 3 to 3.5 m^2^ g^–1^ for Ir^3+^:BTO compared to Ir^4+^:BTO. This minor increment (factor 1.2) in the surface area cannot explain 2 orders of magnitude increment in the H_2_ evolution activity observed for Ir^3+^:BTO compared to Ir^4+^:BTO. Thus, the data presented in Figure 6 can indeed be attributed to the changes in the valence state of Ir, which is explained as follows. The partially occupied Ir^4+^ in Ir^4+^:BTO acts as deep traps (T_2_ transition in Figure 4d) for electrons and thus is expected to reduce the electron lifetime. Consequently, electron transport toward the water interface for H_2_ generation will be inefficient. A similar observation was noticed in the Rh^4+^-doped SrTiO_3_.^19^ To further study this behavior in Ir-doped BTO, an electrochemical impedance spectroscopy analysis was conducted. Electrochemical impedance spectra in Figure S8 illustrate a larger semicircle for Ir^4+^:BTO, which is indicative of higher charge transfer resistance compared to Ir^3+^:BTO. This would further impede electron transport towards the surface of the photocatalyst to realize H_2_ evolution in Ir^4+^:BTO. This notion is supported by observing lower photocurrent density for Ir^4+^:BTO discussed earlier in Figure 5. Moreover, the energetic position of Ir^4+^ is thermodynamically not favorable to realize H_2_ generation. All of these reasons collectively lead to H_2_ evolution of 0.008 μmol for Ir^4+^:BTO under visible light (λ > 400 nm).

Ir^3+^:BTO showed 2 orders (0.81 μmol) of magnitude enhancement in H_2_ generation than Ir^4+^:BTO, which is rationalized as follows. For Ir^3+^:BTO, the concentration of electron-trapping Ir^4+^ levels is decreased, and concurrently, the T_3_ transition (Figure 4d) is pronounced that promotes electrons to the CB instead of trapping to Ir^4+^ levels. Both of these effects are expected to collectively contribute in extending the electron lifetime for Ir^3+^:BTO compared to Ir^4+^:BTO, which consequently favors electron transfer to enhance the H_2_ generation yield. A similar trend in valence state-dependent H_2_ evolution activity was observed (Tables S2 and S3) when measured under UV light and for Ir-doped BTO with a higher doping level. These results unambiguously demonstrate the crucial role of the Ir valence state in realizing pronounced visible light-induced H_2_ generation.

In the work presented here, the emphasis is to reveal the origin of valence state-dependent optoelectronic properties, photoelectrochemical response, and photocatalytic H_2_ evolution generation efficiency. We are currently in the process of identifying parameters to enhance the H_2_ evolution activity further, particularly in λ > 500 nm. Furthermore, we are planning to construct a z-scheme photocatalyst with this photocathode material. Besides photocatalytic applications, realizing optical control over the Ir valence state may also find application in other fields such as spintronics and optoelectronics.

## 4 Conclusions

The H_2_-TPR experiment selectively and efficiently converted Ir^4+^ to Ir^3+^ in Ir-doped BTO photocatalysts. The origin of Ir valence state-dependent photocatalytic H_2_ evolution efficiency was revealed using a range of complementary spectroscopy tools and computational analysis of the electronic structure. The Ir^3+^:BTO, which showed an increment of 2 orders in photocatalytic H_2_ generation and a factor of ≈2.3 enhancement in Faradaic efficiency recorded during chronoamperometry relative to Ir^4+^:BTO, had filled in-gap Ir^3+^ donor levels, allowing optical transition to the CB, populating the CB with free electrons. In contrast, partially occupied Ir^4+^ levels in Ir^4+^:BTO acted as a sink for photogenerated electrons, thus reducing the number of free electrons in the CB available for H_2_ evolution. This work established a comprehensive correlation among the energetic position of the dopant, its electron occupancy, the resulting optoelectronic properties, and photocatalytic H_2_ evolution activity. These results help in designing photocatalysts to efficiently harness low-energy visible/NIR photons to realize enhanced photocatalytic activity. Collectively, the promising features of Ir^3+^:BTO revealed in this work demonstrate its potential as a photocathode for visible light-induced *z*-scheme reactions.

## Supporting Information Available

XPS spectra of Ti and Ba, DOS of Ir^3+^:BTO and Ir^4+^:BTO, H_2_ evolution monitored during chronoamperometry, and Faradaic efficiency and electrochemical impedance spectra of Ir^3+^:BTO and Ir^4+^:BTO (PDF)
