# Supplementary material for: Unveiling Valence State-Dependent Photocatalytic Water Splitting Activity and Photocathodic Behavior in Visible Light-Active Iridium-Doped BaTiO3
Source: ACS Appl Mater Interfaces. 2024 Feb 8;16(7):8763–71. doi: 10.1021/acsami.3c16710 (PMC10895576; doi:10.1021/acsami.3c16710)
Supplement: Supplementary file 1 — am3c16710_si_001.pdf [file am3c16710_si_001.pdf]

Supporting Information

Unveiling Valence State-Dependent Photocatalytic Water Splitting Activity and Photocathodic Behavior in Visible-light Active Iridium-doped $BaTiO_3$

Sujana Chandrappa[1], Stephen Nagaraju Myakala[2], Namitha Anna Koshi[3], Simon Joyson Galbao[1], Seung-Cheol Lee[3], Satadeep Bhattacharjee[3], Dominik Eder[2], Alexey Cherevan[2] and Dharmapura H. K. Murthy[1,4]*

1. Department of Chemistry, Manipal Institute of Technology, Manipal Academy of Higher Education, Manipal, Karnataka, India-576104.
   *Email: murthy.dharmapura@manipal.edu

2. TU Wien, Institute of Materials Chemistry, Getreidemarkt 9/BC/02, 1040, Vienna, Austria.

3. Indo-Korea Science and Technology Center (IKST), Korea Institute of Science and Technology, Bengaluru 560064, India.

4. Center for Renewable Energy, Manipal Institute of Technology, Manipal Academy of Higher Education, Manipal, Karnataka, India-576104.

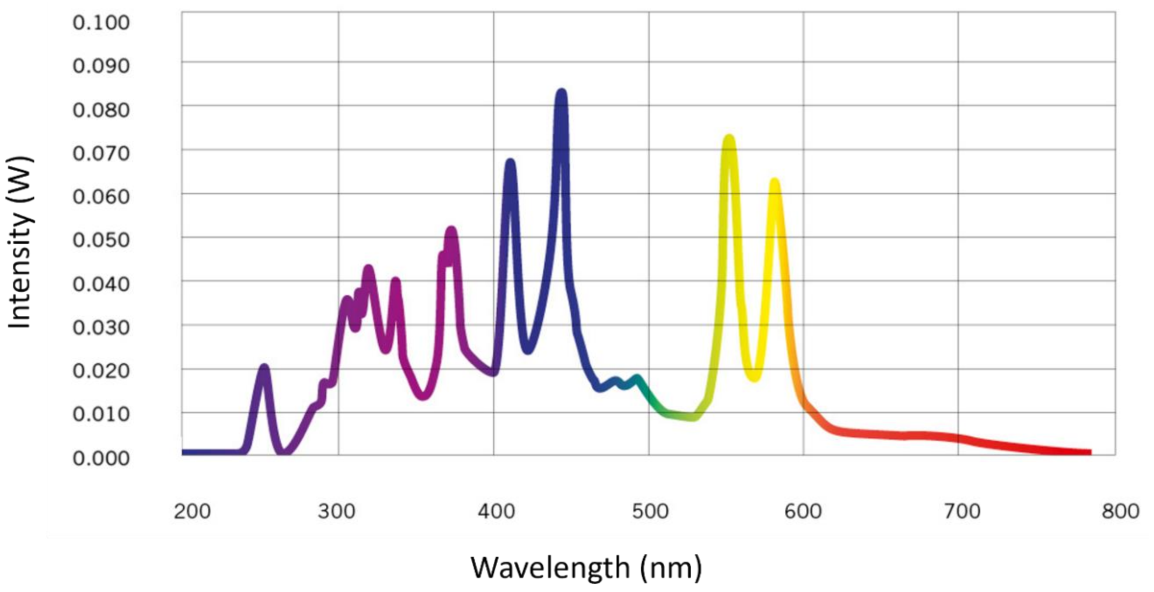

Figure S1: Spectral distribution of the Hg lamp (light source) used for the photocatalytic H_2 evolution reaction.

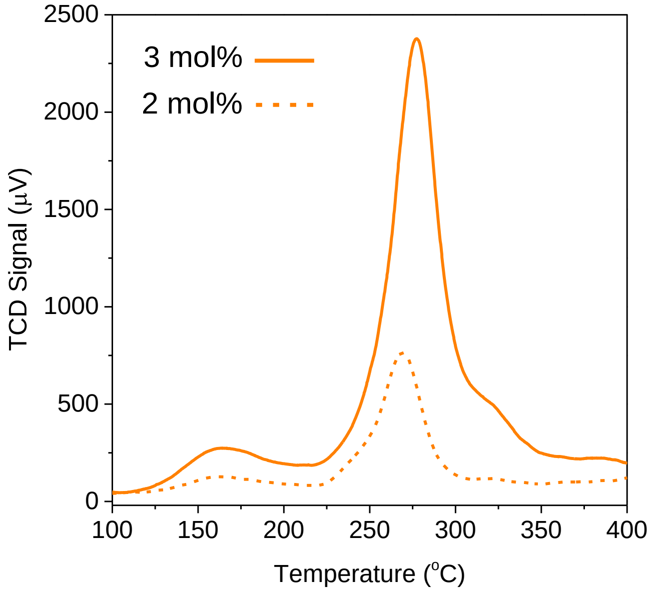

Figure S2: H_2-TPR profiles of Ir-doped BTO photocatalysts with different doping levels.

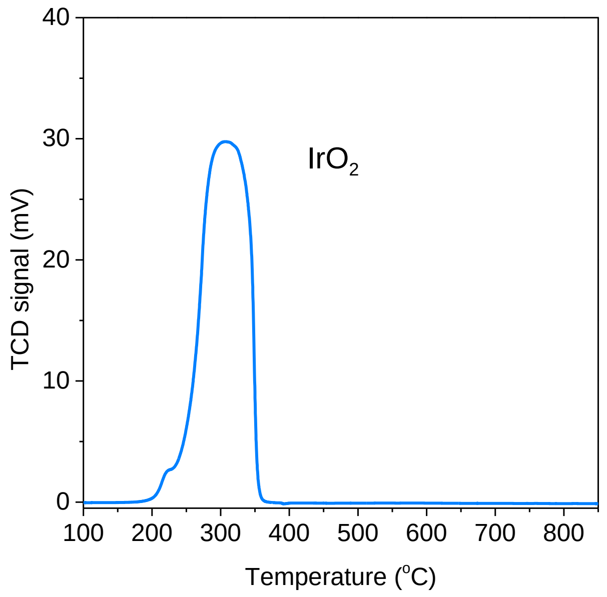

Figure S3: H_2-TPR profile of pristine IrO_2 measured up to 850 °C. Note that minor differences in the shape of the H_2-TPR profiles of Ir^{4+}:BTO and IrO_2 can be explained by considering differences in the chemical bonding behaviour (and environment) of Ir in IrO_2 and the Ir^{4+} in Ir-doped BTO host lattice. Such effects will naturally yield minor variations in the temperature required for the reduction reaction.

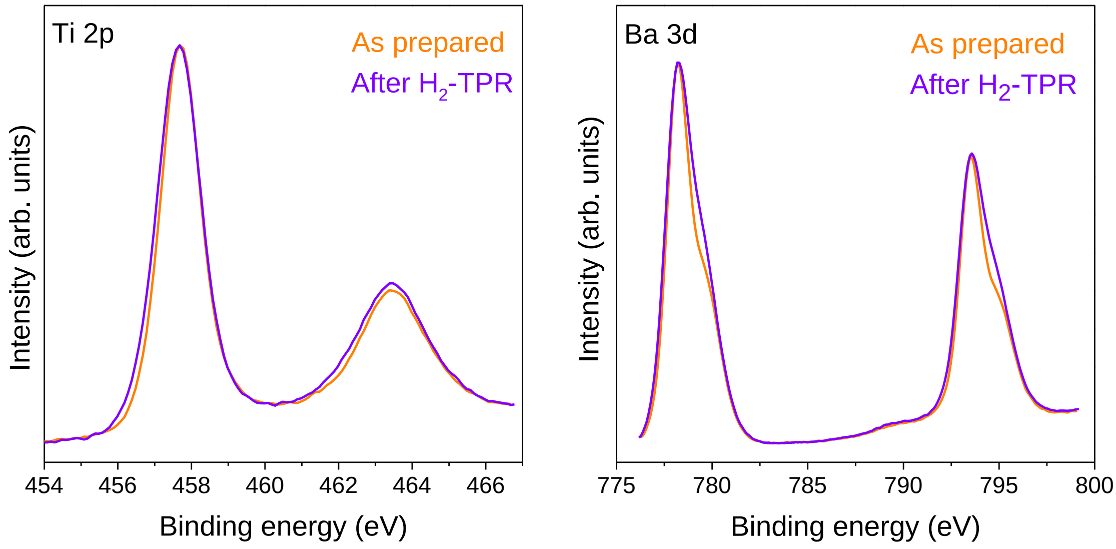

Figure S4: High-resolution Ti 2p and Ba 3d core level spectra of as prepared Ir^{4+}:BTO sample and Ir^{3+}:BTO formed after H_2-TPR experiment. Noticing <0.1 eV peak shift, unlike 1.3 eV shift for Ir 4f indicates that H_2-TPR experiment selectively converted Ir^{4+} to Ir^{3+}.

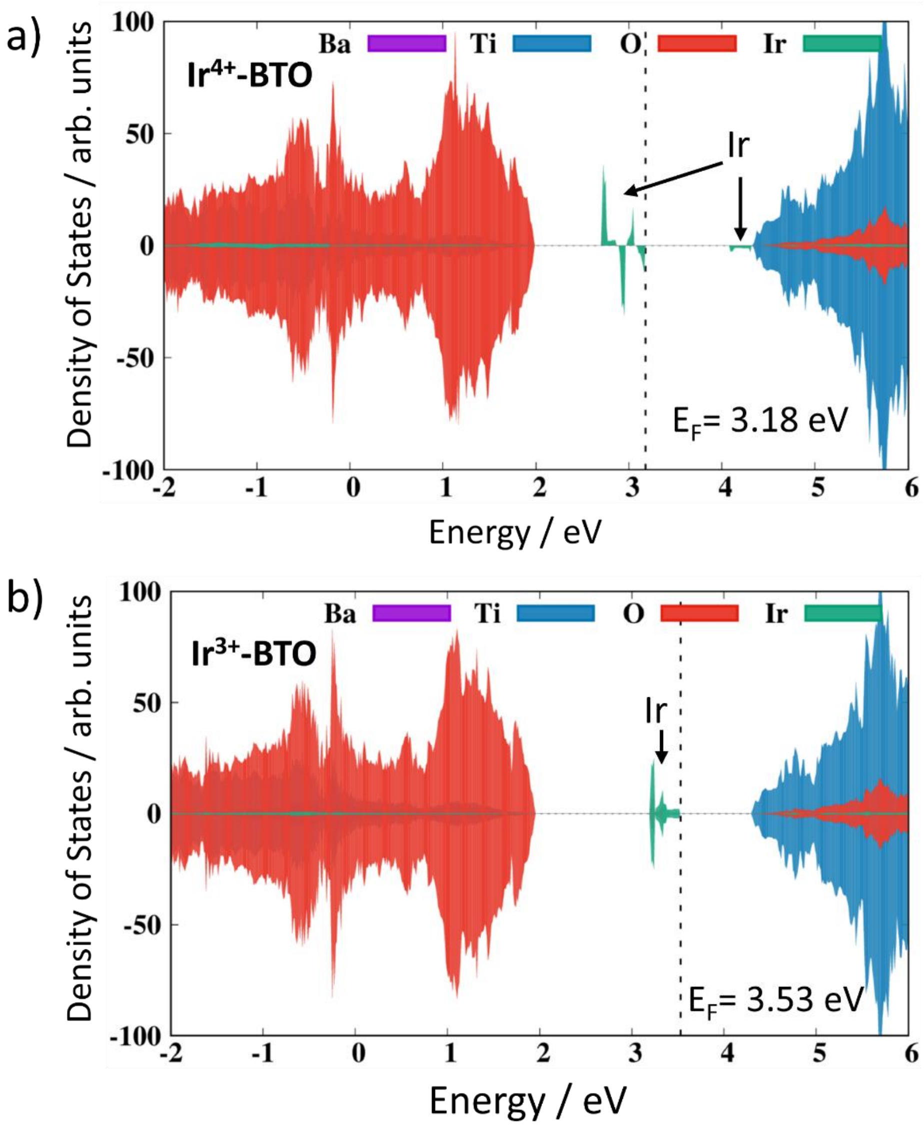

Figure S5: DOS of a) Ir^{4+}:BTO and b) Ir^{3+}:BTO with Fermi level indicated by dotted line. The E_F of Ir^{4+}:BTO is located at 3.18 eV while that of Ir^{3+}:BTO moves further towards the CB and is situated at 3.53 eV. The upshifting of E_F and the absence of partially occupied Ir 5d orbitals between the E_F and the CB for Ir^{3+}:BTO suggests the filling up of the Ir in-gap states, thus suggesting increased DOS of Ir^{3+} states.

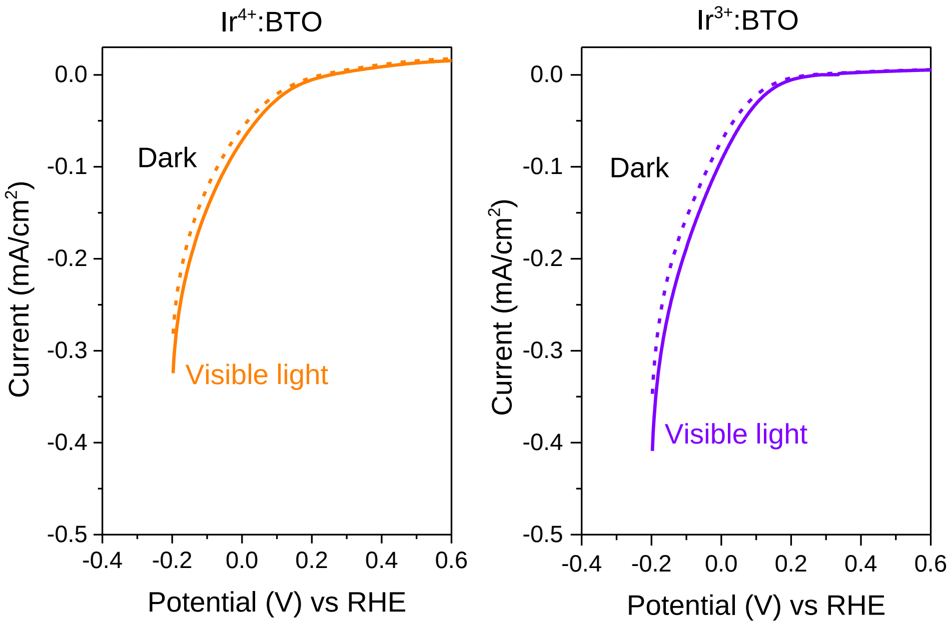

Figure S6: Linear sweep voltammograms of Ir^{4+}: BTO and Ir^{3+}:BTO with 4 mol% doping , under dark and visible-light irradiation (λ> 420 nm) irradiation.

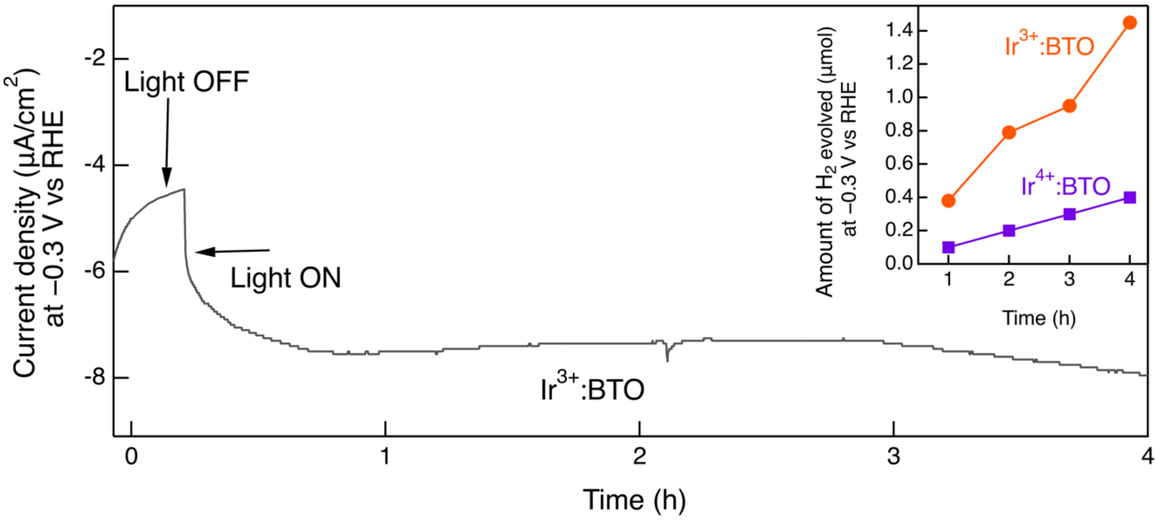

Figure S7: Chronoamperometric measurement of Ir^{3+}:BTO conducted at −0.3 V vs RHE. H_2 evolution from Ir^{3+}:BTO and Ir^{4+}:BTO quantified for four hours at an interval of 1 hour is depicted in the inset.

Chronoamperometric measurements depicted in Figure S7 is conducted in 0.1 M K_2SO_4 using the three-electrode system, i.e., platinum plate as counter electrode, photocatalyst drop-cast FTO (fluorine tin oxide) coated glass substrate (resistance: 7 ohm/sq) with drop cast area of 2x1 cm^2 as working electrode and Ag/AgCl in 3M KCl as reference electrode. 5 mg of

photocatalyst (Ir^{4+}:BTO and Ir^{3+}:BTO) was added to the solution containing 120 μL of distilled water, 100 μL of isopropanol, 5 μL of dimethyl formamide, and 5 μL of Nafion. The solution was sonicated for 45 minutes to prepare the photocatalyst ink. 200 μL of the prepared ink was drop-cast on the FTO-coated glass substrate. The apparent area of electrode containing the photocatalyst was 2 cm^2 area. The electrodes were dried under an infrared lamp and calcined at 300 °C for 3 hr. Potential vs Ag/AgCl is converted to potential vs RHE and is represented in Figure S7. The air-tight photoelectrochemical cell was purged with argon gas for 30 minutes before the measurements to exclude any dissolved oxygen. Chronoamperometric measurements were conducted in the dark for 20 minutes followed by visible light (420-700 nm) irradiation for 4 hours at −0.3 V vs RHE. H_2 evolved during chronoamperometry under visible light irradiation was quantified by gas chromatography (Shimadzu GC-2030 equipped with a barrier discharge ionization detector). The above recorded current density is lower than that discussed in Figure 5c. This can be attributed to the change in the substrate from ITO in Figure 5c to FTO in Figure S7 having a higher resistance. Furthermore, the apparent area of the electrode containing the photocatalyst in Figure 5c is 0.25 cm^2 whereas that discussed in Figure S7 is 2 cm^2. Hence, the current density (current/unit area) in the latter is expected to be less than former.

Figure S7 represents the chronoamperometric data of Ir^{3+}:BTO and the H_2 evolution recorded during the chronoamperometry is monitored for 4 hours and is depicted in the inset. Hence, the observed photocurrent is indeed due to photoelectrochemical H_2 evolution from water-splitting. The Faradaic efficiency of Ir^{3+}:BTO and Ir^{4+}:BTO calculated at the end of 4 h was found to be 75.8% and 32.7%, respectively. Hence, the H_2 evolution (given in the inset) and subsequently, the Faradaic efficiencies of Ir^{3+}:BTO and Ir^{4+}:BTO clearly demonstrate the effect of Ir-valence state on the photoelectrochemical activity.

Table S1: Surface area determined for Ir-doped photocatalysts with different mol%.

Sl. No	Amount of doping (mol%)	Surface area ($m^2\ g^{-1}$) Ir^{4+}:BTO	Surface area ($m^2\ g^{-1}$) Ir^{3+}:BTO
1.	2	3.0	3.5
2.	4	2.5	2.3

Table S2: H_2 evolved using 2 mol% Ir-doped BTO photocatalysts under the ultraviolet (UV) light.

photocatalysts	H_2 evolved (µmol)
Ir^{4+}:BTO	0.015
Ir^{3+}:BTO	1.88

Reaction conditions: 1 wt% Pt cocatalyst loading, 100 mg phtotcatalysts, 10 vol% aqueous methanol, 24 h.

Table S3: H_2 evolved using 4 mol% Ir-doped BTO photocatalyst.

photocatalysts	UV light (λ<400 nm) (µmol)	Visible light (λ>400 nm) (µmol)
Ir^{4+}:BTO	0.0035	0.002
Ir^{3+}:BTO	0.07	0.226

Reaction conditions: 1 wt% Pt cocatalyst loading, 100 mg phtotcatalysts, 10 vol% aqueous methanol, 24 h.

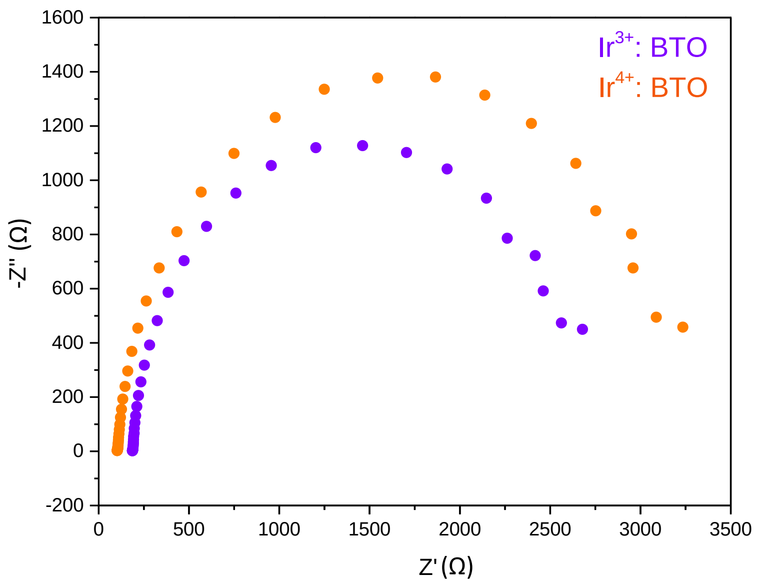

Figure S8: Electrochemical impedance spectra of Ir^{3+}:BTO and Ir^{4+}:BTO.

Electrochemical impedance spectroscopy measurements were recorded in Metrohm Autolab PGSTAT 204 potentiostat using 0.1 M K_2SO_4 as electrolyte with RHE as reference electrode, platinum counter electrode and catalyst coated ITO substrate as working electrode. The

measurements were recorded at DC potential of 0 V vs RHE in the frequency range of 5000 Hz to 100 mHz at an amplitude of 10 mV.
